# Awareness and Knowledge of the Effect of Ultraviolet (UV) Radiation on the Eyes and the Relevant Protective Practices: A Cross-Sectional Study from Jordan

**DOI:** 10.3390/healthcare10122414

**Published:** 2022-11-30

**Authors:** Mohammad A. Alebrahim, May M. Bakkar, Abdulla Al Darayseh, Aya Msameh, Dana Jarrar, Saja Aljabari, Walla Khater

**Affiliations:** 1 Department of Allied Medical Sciences, Faculty of Applied Medical Sciences, Jordan University of Science and Technology, Irbid 3030, Jordan; 2 Emirates College for Advanced Education, Abu Dhabi P.O. Box 126662, United Arab Emirates

**Keywords:** ultraviolet (UV) radiation, ocular damage, protective eyewear, Jordan

## Abstract

*Background:* Overexposure to ultraviolet (UV) radiation is linked to serious adverse health effects that are cumulative in nature and affect children more than adults. UV radiation has also been reported to have serious complications for the eye, particularly in areas with a high UV radiation index. Increasing public awareness about the harmful effects of UV radiation on the eye and promoting awareness about protection against UV radiation may prevent eye disease related to UV radiation damage and help in the improvement of public health in general. This study aims to assess public awareness and knowledge of UV radiation and practices toward UV protection in Jordan, which is a country recognized as having a relatively high UV index throughout the year. *Methods:* A cross-sectional study was performed using an online questionnaire using Google Forms^®^ to assess people’s awareness, knowledge, practices toward eye protection from UV radiation, and the reasons for not wearing UV-protective eyeglasses in Jordan. Sociodemographic information of participants including age, gender, education level, and employment status was also acquired. People’s knowledge on UV protection and harmfulness was measured via rewarding their correctly answered knowledge questions with one mark and zero for incorrectly answered questions based on key answers defined from the literature. *Results:* A total of 1331 participants (77% females and 23% males) with an average age of 26(±10) years completed the online questionnaire. Participants showed generally high levels of knowledge and awareness about UV radiation and its harmful effects. Nevertheless, participants showed a low level of knowledge about the link between UV radiation and some of the ocular diseases in the questionnaire. Practices toward UV radiation protection where inadequate, with 59% of the respondents reporting that they do not use any protective eyewear from natural UV radiation. The main reported reason for not wearing UV-protective sunglasses was uncertainty in the efficiency of UV protection in sunglasses, as reported by 47% of the participants who do not wear UV-protective sunglasses. *Conclusions:* The awareness of UV radiation and its harmful effects is high in the studied population. Participant knowledge is also relatively high in relation to nature of solar UV radiation, other synthetic sources of UV radiation, and the most dangerous UV exposure time. However, low participant knowledge was measured on the association between UV radiation with ocular disease and the role of UV-protective eyeglasses. Participant practice toward UV radiation protection was found to be insufficient. Thus, it is important to further increase the knowledge of damaging effects of solar and synthetic UV radiation and emphasize the benefits of eye protection from UV radiation. Eye care practitioners should target youth by different strategies including health campaigns, media, and clinics.

## 1. Introduction

Ultraviolet (UV) radiation is a kind of solar radiation with a wavelength that ranges from 100 nm to 400 nm and is divided into three parts: UV-A (315–400 nm), UV-B (280–315 nm), and UV-C (100–280 nm), where the shorter the wavelength, the more harmful the effects [1,2]. All the UV-C and up to 95% of UV-B do not reach the earth because the ozone layer absorbs them. Therefore, only 5% of UV-B and 95% of UV-A are transmitted to the land [3]. Sunlight is considered the primary source of UV radiation. On the other hand, there are many secondary sources for UV radiation, such as suntanning beds, electric sparks, photographic flood lamps, welding arcs, and halogen desk lamps [3,4,5].

UV radiation is not a part of the visible light spectrum and is not perceived by the visual system. Therefore, its harmful effects cannot be felt until tissue damage has developed [6]. The harmful effect of the radiation on human health increases with excessive cumulative exposure to the UV radiation sources [7] and may be associated with tissue atrophy, skin pigmentation changes, wrinkles, and malignancies, including melanoma and basal cell carcinoma [1,8].

Scientific evidence shows that acute, large-dose exposure to UV radiation can cause serious ocular complications such as photokeratitis and photo conjunctivitis. Further, exposure to small doses of UV, specifically UVB, has been reported as a risk factor in developing several ocular diseases including cataract, pinguecula, pterygium, and squamous cell carcinoma of the cornea and the conjunctiva [9,10,11]. Unprotected exposure to UV rays can possibly cause significant ocular damage in young children compared to adults, due to the relatively large pupil size and the more transparent ocular media in children [12]. Hence, it is suggested that up to 80% of a person’s lifetime exposure to UV radiation is reached before the age of 18 [13]. Thus, it is necessary to provide eye protection from UV radiation at a young age.

There are several ways of protecting the eye from UV radiation to avoid possible ocular damage caused by excessive exposure. The most common method is the use of sunglasses, which are eyewear that contain a UV-protection filter which filters 99–100% of UV radiation [14]. These types of UV-blocking sunglasses can be supplementarily designed by adding a “wrap-around” frame and using sideway shields on the frame to reduce the entry of UV radiation into the eye [14,15]. Other recognized methods to protect the eyes from UV radiation include contact lenses [16] and implanted intraocular lenses that contain UV-blockers [15,17].

Several studies have been published to evaluate the knowledge and protection behaviors concerning UV radiation among populations. However, to date, there are a lack of studies that assess awareness levels regarding UV radiation in relation to ocular damage among people in Jordan. Jordan is a Mediterranean country labelled as having a high intensity of UV radiation, known as UV index (UVI), for most of the year and with a maximum UVI level of 12 when measured in the summer season [18]. Therefore, this study aims to assess the knowledge and public attitudes regarding UV radiation and ocular protective behaviors in Jordan. The outcomes of the current study essentially recommend increasing public awareness of possible UV-related ocular damage and alert the public and healthcare providers to enhance and apply protection strategies for UV exposure.

## 2. Methods

### 2.1. Study Design and the Questionnaire

The study is a cross-sectional design that used a structured web-based questionnaire which was designed and modified based on a previous questionnaire developed by Lee et al. [19]. The questionnaire was intended to be conducted in Arabic. The comprehension and scientific appropriateness of the Arabic questionnaire was initially reviewed by a focus group consisting of three faculty members in the department of Allied Medical Sciences at the Jordan University of Science and Technology (J.U.S.T). The revised version of the questionnaire was then administered to 20 participants in order to check the respondents’ comprehension of the questions and the required response time. Responses from the pilot sample were not included in the final data analysis.

The final version of the questionnaire showed very good psychometric properties including an acceptable internal consistency and test-retest reliability (interclass correlation coefficient, r = 0.832, *p* < 0.001). The construct validity for the questionnaire was also high.

The final Arabic version of the questionnaire was administered online using Google Forms^®^ (Google Inc., Mountain View, CA, USA) and was shared via social media, WhatsApp, and LinkedIn for a period of 4 weeks between July 2021 and August 2021. The estimated time needed to answer the questionnaire was approximately 8 min on average.

Questions included in the questionnaire were divided into five sections: (1) the sociodemographic information of participants including age, gender, education level, and employment status; (2) awareness regarding UV radiation (sources, harm, etc., and importance of wearing UV-protective eyeglasses; (3) knowledge of UV radiation; (4) practices toward eye protection from UV radiation (solar UV and artificial sources of UV); and (5) reasons for not wearing UV-protective eyeglasses.

### 2.2. Data Analysis

Data were analyzed using the Statistical Package for Social Sciences software version 21 (SPSS, International Business Machine Corp. IBM, Chicago, IL, USA). The mean and standard deviation were used to describe continuously measured variables. The frequency and percentages were used for categorically measured variables. The histogram and the statistical Kolmogrove–Smirnove test were used to assess the normality assumption of continuous variables and Levene’s test of homogeneity of variance for testing the equality of statistical variance assumption. The multiple response dichotomies analysis used for variables with more than one option (e.g., seasons of eyeglasses).

People’s knowledge on UV radiation was measured via rewarding their correct answers to knowledge questions with one mark (point) and with zero for incorrect answers. First, a total UV protection knowledge score was computed by adding up people’s marked answers to the thirteen knowledge questions yielding a total UV protection knowledge score between 0–13 marks. Next, this score was transformed into a percentage via dividing people’s knowledge score by the maximum possible score (=13), then multiplying the yielded product by a hundred.

People’s UV knowledge score was dichotomized into low and high knowledge scores based on the sample median knowledge score (median = 9 points out of 13). Then, a multivariate binary regression analysis was used to assess the statistical significance of the predictors of people’s odds of having a high UV-protection knowledge, and their odds of not wearing UV-protective eyeglasses, the association between the people’s socio-demographic, and other relevant predictor variables with the analyzed UV knowledge; protection behaviors were expressed as an odds ratio with an associated 95% confidence interval. The alpha significance level was considered at a level of 0.05.

### 2.3. Ethics Approval

The study was approved by the Institutional Review Board (IRB) at the Jordan University of Science and Technology (Irbid, Jordan). The ethical reference number was: 25/142/2021.

The aim and importance of the study was explained to the participants. Informed consent was obtained electronically from all participants before proceeding to the survey questions. Participation in this study was voluntary. To ensure privacy and confidentiality, the anonymity of the participants’ personal information was preserved. The study protocol complied with the provisions of the Declaration of Helsinki regarding research on human participants.

## 3. Results

### 3.1. Participants’ Demographics

A total of 1331 participants (77% females and 23% males) completed the online questionnaire. The mean age (±standard deviation) of the participants is 26 (±10) years with the majority of the study population was in the age group 18–30. Demographic information of all participants is showed in Table 1.

### 3.2. General Awareness Regarding UV Radiation, Association with Ocular Harm, and UV-Protective Eyeglasses

The majority of the respondents (97%) reported that they were aware of UV radiation. Additionally, a high proportion of the participants reported that they were aware that UV radiation could result in harm to the human body and eyes, as shown in Table 2.

Participants were also asked about UV-protective eyeglasses. The results showed that although the majority of the participants had heard about UV-protective eyeglasses, less than half of the participants (48%) agreed that the UV protection featured in eyeglasses is effective in blocking harmful UV rays and should be considered in any eyeglasses. A total of 49% reported that they do not have enough information about how UV protection works and about its effectiveness, and 4% of them believed that UV protection is a marketing scam used to increase sales and profit of marketed eyeglasses.

### 3.3. Knowledge of UV Radiation, Its Harm for the Human Eye, and Methods of Protection

Participants were asked to answer thirteen questions measuring their knowledge related to UV radiation, its harm for the eye, and methods of protection. The results from the analysis of the knowledge assessment of the participants are displayed in Table 3.

Generally, most respondents showed good knowledge regarding UV radiation, the most dangerous UV exposure time during the day, and other synthetic sources of UV radiation. Respondents were also asked about their knowledge about some ocular diseases associated with UV radiation, including cataract, pterygium, pinguecula, and AMD. Respondents were able to define these diseases. However, respondents showed substantial knowledge about the link between UV radiation with the questioned ocular diseases.

### 3.4. Participant Practices toward Protection from UV Radiation

Participants were further asked about their practices toward ocular protection from natural UV radiation emitted from the sun. Responses were distributed as the following: 59% of the respondents reported that they do not use any protective eyewear when they go outside, 30% of the participants reported wearing quality sunglasses with full, tested UV protection bought from optical shops, and 11% of the participants reported that they wear cheap, dark cosmetic sunglasses bought from accessory shops and they are uncertain if they have UV protection.

Participants were also asked whether they use UV radiation protection (eyeglasses or protective screens) to protect their eyes from artificial sources of UV radiation emitting from light-emitting diodes (LEDs), tanning booths, mercury vapor lamps, and electronic devices such as computers and mobile phones. The majority (92%) of participants reported not using any protection from artificial sources of UV radiation.

### 3.5. Multivariate Logistic Regression Analysis of Participants Knowledge of UV Harm and Protection

A multivariate binary logistic regression was performed to assess factors correlated with measured levels of participant knowledge. Table 4 shows that sex is correlated significantly with the odds of having a high UV knowledge; male respondents have a significantly lower (62% times less) UV knowledge level compared to females, *p* < 0.001. Furthermore, the age of participants is correlated significantly and negatively with the odds of having a high UV knowledge. As participant age tends to rise by one year, the likely odds of being highly knowledgeable about UV harm and protection tends to decline by a factor equal to 3% times less, *p* < 0.001. Participant work status and work environment (indoor or outdoor) does not converge significantly on the odds of participants having a high UV knowledge. However, participants with a former awareness about UV rays were found to be significantly more likely (2.4 times more) for a high UV knowledge compared to those with a low level of awareness of UV radiation, *p* = 0.036.

People perceived time of best eyeglass use did not converge significantly on their UV knowledge, but people who use protective eyeglasses during electronic device use were significantly more likely to UV high knowledge than those who do not wear such protection on average, *p* = 0.047. In addition, people previously made aware of the importance of eye UV-protective measures were significantly more likely for high UV knowledge than others unaware of the usefulness of the eye protection, *p* < 0.001.

The analysis model findings showed that people who were keen to buy eyeglasses with UV protection were found to be significantly more likely (1.4 times more) to have a high UV knowledge compared to people who are not keen to buy eyeglasses with UV protection, *p* < 0.001. Nonetheless, people who believed in the effectiveness of UV-protective features in eyeglasses were found to be significantly more (1.7 times more, *p* < 0.001) likely to have had a high knowledge of UV harmfulness and protection compared to others who believed the UV protection in sunglasses is a marketing scam or those who do not even use eyeglasses. Interestingly, people who bought their eyeglasses from optics shops were found to be significantly more likely (2.0 times higher, *p* < 0.001) to have a high UV knowledge on average compared to those who buy eyeglasses from accessory shops or those who had never bought them. However, people who advised that they do not wear protective eyeglasses or any eyeglasses with featuring UV-protection were found to be significantly less likely (39% times less, *p* = 0.001) for having a knowledge of high UV harmfulness and protection on average compared to those who do use protective eyeglasses or features of UV protection in general.

### 3.6. Reasons for Not Wearing UV-Protective Sunglasses

Respondents who do not wear any protective sunglasses (790, 59% of the study population) were further questioned about their chief reason for not wearing UV-protective eyeglasses. A total of 7% of the respondents reported that they have never heard about UV-protective eye glasses; 18% reported that the appearance and weight of sunglasses on the nose and face is the main reason for not wearing sunglasses; 14% of the respondents stated that the high price of quality sunglasses prevents them from buying protective eyeglasses; 14% of respondents suggested that UV-protective sunglasses affected their perception of colors; and 47% of the participants reported that they do not believe in the efficiency of the UV protection of sunglasses.

A further statistical analysis was performed to study factors affecting people’s choice for not wearing protective eyeglasses, as shown in Table 5. First, the use of protective eyeglasses was dichotomized as follows (0 = wears UV-protective eyeglasses and 1 = does not wear UV-protective eyeglasses); this outcome was then regressed using the multivariate logistic binary regression model against people’s socio-demographic characteristics and their practices as well as knowledge of UV and other UV protection attitudes.

The results from the multivariate analysis model showed that people’s sex correlated significantly with their odds of not using UV-protective eyeglasses. Males were significantly more likely (1.4 times more, *p*-value = 0.046) for not using UV-protective eyeglasses than female respondents on average. Moreover, people’s age converged significantly, *p* < 0.001, but negatively on their odds of not using UV-protective eyeglasses. Denoting that as people’s age tended to rise by one year, their odds of lacking UV protection declined by a factor equal to 2% times less on average.

The analysis model also showed that retired people were significantly more likely (1.6 times more, *p* = 0.002) to not use UV-protective eyeglasses than people in other occupations. Furthermore, university students were found to be significantly more likely (1.7 First, p9 times more, *p* = 0.02) to not use UV-protective eyewear as well. However, respondents who bought their eyeglasses from opticians were found to be significantly less (85% times less) likely to lack UV protection compared to people who bought their eyeglasses from accessories shops and those who do not use protective eyeglasses in general <0.001. Moreover, the multivariate analysis model showed that people’s UV protection and harm knowledge mean score has correlated significantly but negatively with their odds of not using UV-protective eyeglasses, as people’s knowledge score tended to rise by 1% on average their likely odds of not using the protective eyeglasses declined by a factor equal to 1.5% times less, *p* = 0.001.

## 4. Discussion

Ultraviolet radiation is considered a main cause of ocular surface disease and cataract, especially in geographical areas with high UVI. Jordan is labeled as a country with a relatively high UVI, with an average UVI of 11 on most days of the year [18]. This study provides an insight into the awareness, the knowledge, and the attitude of people in Jordan toward UV radiation harm to the eye as well as UV protection. Awareness merely refers to an understanding of the general information on a certain topic. Thus, in the current study, it was substantially required to assess people’s knowledge of UV harm and the methods of protection.

The current study reports a good level of awareness of UV radiation, its harmful effects on the eyes, as well as the usage of UV-protective eyewear. However, participants showed substantially moderate awareness of the important purpose of the UV protection feature in eyeglasses. Our findings were comparable to those reported by other countries, where studies also showed a high level of public general awareness of UV radiation and its harm. To mention a few, a study in Saudi Arabia reported that the majority of the population have heard about UV radiation and its associated ocular diseases [20]. Similarly, in a study in South Africa, most participants knew that sunlight has adverse effects on the eyes [21]. Likewise, a high level of awareness was reported in Australia regarding exposure to ultraviolet radiation when engaging in outdoor activities [22].

In the second part of the study, we assessed participants’ knowledge of UV radiation harm and protection against it more specifically on the basis of a number of questions that measured in-depth knowledge about the topic. The overall estimated level of knowledge was found to be average (51.38%) among the residents of Jordan. The research sample showed their apprehension of the different sources of UV radiation such as natural solar UV radiation and synthetic sources of UV radiation as well. They also showed awareness about the most dangerous times of the day and season/s of the year to be exposed to UV radiation, when UV eye protection is highly recommended. However, respondents were unaware of the role of UV-protective eyeglasses in preventing eye disease and damage caused by UV radiation and the greater consequences of UV radiation on younger people in comparison to older people. Additionally, a substantive proportion of the respondents lacked knowledge about the link between UV radiation and specific ocular diseases. Furthermore, the majority of the respondents could not differentiate that commercial (cheap) sunglasses may pose a risk to human eyes due to a lack of UV-protective features. This may be attributed to the deficient medical background of participants and low exposure of medical material displayed by various media platforms. Another explanation of low levels of knowledge about harm from UV radiation among the study population may also be linked to the low prevalence of sun-related skin cancers in Jordan [23], as high levels of UV knowledge have been reported in populations of countries with higher incidence of skin cancer [24].

Similar to our findings, low levels of knowledge on the relation of UV radiation and ocular harm were also reported in many countries. A study in Northeast China reported that whereas the majority of the studied population knew the harmful effect of UV radiation on the skin, a low percentage of people could identify the harmful effect of UV radiation on the eye [25].

A further analysis reveals that females have more knowledge of UV harm and protection. This may be explained by the interest of women in self-care and body image that is promoted by society and the media [26]. Predictably, participants who are more aware of UV radiation were found to significantly have more UV radiation related knowledge compared to those with less awareness of UV radiation. Moreover, it was found that age of the participants negatively correlated with level of knowledge in the study.

In regard to preventive measures taken toward ocular UV protection, only a third of the respondents reported using certified UV-protective eyewear. Whereas the majority of the study population do not wear UV-protective eyeglasses at all, or they wear low quality sunglasses with no certified built-in UV filter. Additionally, the majority of the participants reported not using protective eyewear for artificial sources of UV radiation. Similar levels of adherence to wearing protective sunglasses was also reported in Melbourne, Australia (36%) [27], Florida, USA (27%) [28], and Northwest Ethiopia [29]. Even lower level of wearing protective sunglasses was reported in Shenyang, China (9%) [25]. In contrast to our finding, 80% of adults in Kuwait [30] and 60.2% in Saudi Arabia [20] reported using protective sunglasses.

In the current study, a further analysis showed that people who are adherent to wearing eyeglasses with UV protection significantly also had high UV-related knowledge. Thus, the lower motivation of the participants toward wearing protective sunglasses may be attributed to low levels of participants’ knowledge about UV radiation-associated harm. Furthermore, females were found to be more compliant toward wearing UV-protective eyeglasses. This may be explained by the fact that women are more informed about skin care and sun protection forms including sunscreen products and protective sunglasses via women’s magazines and media [24]. The finding that women are more aware, knowledgeable, and adherent to sun protection compared to men has also been reported by many studies [24,31,32,33]. Surprisingly, older people in this study were found to be less adherent to using protective eyeglasses. This contradicts the fact that elderly people should take more precautions as they are more susceptible to sun-related ocular conditions such as cataract [34].

The high price of quality UV-protective sunglasses could be a possible reason for refraining from the use of protective sunglasses in the study population. The price of the sunglasses usually increases in brands that use good quality material and innovation to enhance maximum protection and durability. Furthermore, the practice of using protective sunglasses may depend on the nature of the profession as those who work and stay indoors may have a lower interest in using protective sunglasses as such people may spend less time outdoors.

To the best of our knowledge this is the first study to assess people’s awareness and knowledge on the exposure to UV radiation and their preventive behaviors against UV radiation in Jordan.

The study used an online survey, which may have resulted in self-report bias, as participants are often biased when reporting their personal opinions and attitudes. Furthermore, there is a large difference in the number between female and male participants that does not match with the female-to-male ratio of the national population in Jordan. This is an expected bias because of a lack of random sampling when using the online survey. Furthermore, the questions used in the questionnaire were closed-ended with one or two choices to select from, which might cause a bias in the responses. Future work is recommended to reveal a more in-depth insight of the level of knowledge about UV radiation and precise protective behaviors. This could be performed using qualitative research methods using semi-structured individual interviews, with a smaller sample size being recruited. Finally, the study investigated the level of knowledge and practices of the participants and related them to influencing factors such as age, gender, and occupation. Albeit it is recommended in a future work to study participants’ perception of possible barriers for not using UV-radiation-protective sunglasses such as the cost of quality sunglasses, cultural and social beliefs about sunglasses that they may hide the wearer’s feelings and beauty features, or if wearing sunglasses may be considered as disrespectful behavior when covering the eyes to avoid eye contact with other people.

## 5. Conclusions

Exposure to UV radiation is a risk for ocular disease. This study assessed the awareness, knowledge, and protective behaviors of UV radiation and its associated ocular harm, and also assessed the influencing factors such as age, gender, and occupation. The study revealed a good level of awareness; however, knowledge of UV radiation was poor in relation to its associated ocular harm and means of protection. Knowledge and protection from UV radiation were positively associated with people of a younger age and in females. These findings suggest that more efforts are required to enhance knowledge and promote the use of protective sunglasses besides using other methods of protection from UV radiation such as hats and umbrellas. This could be promoted at a younger age through health education in schools and health-promotion campaigns through media and by ophthalmologists to reduce the risk of ocular damage due to excessive unprotected exposure to UV radiation.

## Figures and Tables

**Table 1:** Participant characteristics (***n*** = 1331).

Variable	*n* (%)
**Gender**	
Male	302 (23)
Female	1029 (77)
**Age**	
18–25	477 (36)
26–30	560 (42)
31–40	133 (10)
>41	161 (12)
**Work status**	
Employed	227 (17)
Unemployed	242 (18)
Undergraduate student	735 (55)
Postgraduate student	29 (2)
Retired	97 (7)
**Nature of work**	
Outdoor jobs	835 (63)
Indoor jobs	254 (19)
Unemployed	242 (18)

**Table 2:** Participant awareness to UV radiation and protection.

Awareness Questions	*n* (%)
Have you ever heard about UV radiation?	
Yes	1289 (97)
No	42 (3)
Do you think that UV radiation may affect human body?	
Yes	1198 (90)
No	133 (10)
Do you think that UV harms the eye?	
Yes	1080 (81)
No	251 (19)
Have you ever heard about UV protection in eyeglasses?	
Yes	962 (72)
No	369 (28)
What do you think about UV protection in eyeglasses?	
- Effective and should be applied	633 (48)
- I do not have enough information. - It is a marketing scam to increase profit	649 (49)
	49 (4)

**Table 3:** Descriptive analysis of participants’ knowledge of UV radiation, its harm, and methods of protection.

	Knowledge Question	Correct *n* (%)	Incorrect *n* (%)
1	What time of the day is the most dangerous exposure time to the sun without protection? (Morning, Noon, afternoon Evening)	1170 (88)	161 (12)
2	Do you know that there are artificial sources of UV rays beside the sun?	897 (67)	434 (33)
3	Do you know what is cataract/lens opacification is?	885 (67)	464 (34)
4	Do you know that exposure to harmful sun rays (ultraviolet radiation) without protection may lead to cataract?	576 (43)	755 (57)
5	Do you know what pinguecula is (yellowish raised growth on the conjunctiva)?	442 (33)	889 (67)
6	Do you know that exposure to harmful sun rays (ultraviolet radiation) without protection may lead to pinguecula?	188 (14)	1143 (86)
7	Do you know what is Age-related macular degeneration (AMD)?	948 (71)	383 (29)
8	Do you know that exposure to harmful sun rays (ultraviolet radiation) without protection may lead to AMD?	190 (14)	1141 (86)
9	Do you know that ultraviolet radiation has a greater danger to young people than older people?	583 (44)	748 (56)
10	Do you know that wearing UV protective eyeglasses prevents UV harmful sun rays’ entry to the eye	708 (53)	623 (47)
11	In which season the protection against UV radiation is necessary? (You can choose more than one answer) (spring and summer)	833 (63)	498 (37)
12	Do you think that wearing protective eyeglasses in winter is important?	812 (61)	519 (39)
13	Do you know that commercial sunglasses have a negative effect on the eyes?	338 (25)	993 (75)

**Table 4:** Multivariate logistic regression analysis of participant knowledge of UV protection and harm.

	Multivariate Adjusted Odds Ratio	95% C.I. for OR		*p*-Value
		Lower	Upper	
Sex (Male)	0.38	0.28	0.52	<0.001
Age (years)	0.97	0.95	0.98	<0.001
Employment	1.1	0.97	1.2	0.16
Nature of work	1.1	0.81	1.6	0.46
Previously heard of UV	2.4	1.06	5.2	0.036
Time of sunglasses use	1.0	0.82	1.2	0.99
Use of protective eyeglasses during TV/PC use	1.7	1.0	2.7	0.047
Previously aware of ultraviolet ray protection	2.0	1.4	2.7	<0.001
Buys eyeglasses with UV protection	1.4	1.0	1.8	0.044
Believes protective eyeglasses are UV effective/protective	1.7	1.3	2.3	<0.001
Buys eyeglasses with UV protection from optics shops	2.0	1.5	2.7	<0.001
Does not wear eye glasses at all	0.61	0.45	0.82	0.001
Constant	0.15			0.017
Dependent variable = high UV knowledge (no/yes).				

**Table 5:** Multivariate Logistic Regression Analysis of not using UV-protective eyeglasses.

	Multivariate Adjusted Odds Ratio	95% C.I. for OR		*p*-Value
		Lower	Upper	
Sex (Male)	1.4	1.0	1.9	0.046
Age (years)	0.98	0.96	0.99	0.005
Occupation = Retired person	1.6	1.1	2.5	0.020
Occupation = University student	1.7	1.2	2.3	0.002
Buys eyeglasses from optics shops	0.15	0.11	0.19	<0.001
Ultraviolet rays Protection and Harm knowledge score	0.99	0.98	0.99	0.001
Constant	11			<0.001
Dependent outcome variable = Does Not use UV-protective eyeglasses (No/Yes).				

## Data Availability

Data is available by emailing the corresponding author.
